# Quality Improvement Protocol: Improving the Use of Nonpharmacological Pain Management Strategies within the Inpatient Hospital Setting

**DOI:** 10.3390/jcm13061680

**Published:** 2024-03-14

**Authors:** Katherine S. Salamon, Christina Russell, Dara DeVinney, Catherine M. Soprano

**Affiliations:** 1 Nemours Children’s Health, Wilmington, DE 19803, USA; dara.devinney@nemours.org (D.D.); catherine.soprano@nemours.org (C.M.S.); 2 Sidney Kimmel College of Medicine, Thomas Jefferson University, Philadelphia, PA 19107, USA; 3 Aveanna Healthcare, Atlanta, GA 30339, USA; christina.russell@aveanna.com

**Keywords:** quality improvement, nonpharmacological pain management, pediatric

## Abstract

**Background:** There are many nonpharmacological pain management services available to hospitalized youth; however, not all youth are offered these services. Lack of knowledge about resources, difficulty with the referral process, and lack of understanding about nonpharmacological pain management services and when to refer patients are among the main reasons for lack of utilization. Quality improvement (QI) initiatives have grown within hospital settings and can serve to create change in fast-paced environments. **Methods:** The current QI project aimed to pilot an educational program to increase the use of nonpharmacological pain management interventions. Staff located on one floor of a pediatric hospital on the East Coast were selected because of the wide range of patient presentations and likelihood that these youth may present with pain during the hospitalization. Following several incremental changes and multiple PDSA cycles, utilization of nonpharmacological pain management services was assessed. **Results:** Education only did not result in increases in nonpharmacological pain management services ordered. A best practice alert for nursing staff, implemented within the electronic medical record, led to a 50% increase in services ordered. **Conclusions:** These results suggest that to improve use of nonpharmacological pain management strategies, hospital systems may need to consider more than education.

## 1. Introduction

Acute and chronic pain are widespread, complex concerns that influence quality of life and health care utilization and can lead to poor long-term health outcomes [1]. While acute pain is common in the inpatient setting, treatment of pain is often a source of frustration for clinicians, patients, and families [2]. Additionally, poorly managed acute pain can contribute to the development of chronic pain [3]. Guidelines to address acute pain within the inpatient setting often highlight the fact that undertreated pain can lead to poor health outcomes. In addition, a range of pain management strategies is often the most effective [1]. Hyland and colleagues [2] outlined 10 important pieces of knowledge to guide best pain management practices, the first of which is implementation of a holistic approach to acute pain management.

The biopsychosocial approach to acute pain management can be useful in decreasing unnecessary inpatient hospital days, decreasing the burden on hospital staff, and improving patient and family satisfaction. In this model, the biological, psychological, and sociocultural needs of the youth and family are met. To address the biological aspects of pain management, careful consideration of the medications administered is needed [1], and attention should be paid to the overall environment, such as allowing for adequate sleep and inclusion of physical therapy. Psychological interventions consider the individual person. For example, consideration of a youth’s medical history and understanding of the current condition and prognosis are important as these can alter the effectiveness of pain management skills. Inclusion of psychological services as available and nonpharmacological pain management strategies is recommended. Lastly, addressing the needs of the family and the family’s understanding of the admission and pain as well as other social determinants of health are important for effective pain control and health outcomes. Johnson [4] highlighted the role of implicit bias, or unconscious negative assumptions, within pediatric pain management, suggesting that implicit bias may influence clinician decision-making, thus contributing to poor pain management and health outcomes.

Given the intersection of pain and pain management, social determinants of health, and quality of life, addressing adequate pain control within the inpatient setting in pediatric institutions is imperative. One way to build on past work is through the inclusion of nonpharmacological pain management strategies. The National Center for Complementary and Integrative Health defines nonpharmacological pain management strategies as any intervention that aids in reducing pain without the use of medication. [5] These can include physical and occupational therapy, yoga, acupuncture, psychology, distraction, and biofeedback, to name a few. Despite the acknowledgement within research [1] and anecdotal experiences of clinicians, nonpharmacological pain management strategies are not utilized as frequently within the inpatient setting. Doyle and colleagues [6] suggest that lack of education about nonpharmacological pain management strategies may play a role. After training nurses on a subset of strategies in a study conducted by Doyle et al. [6], the use of those specific strategies increased. In another study, Jira and colleagues [7] within an Egyptian hospital found similar results, that knowledge and attitude about these strategies played a significant role in recommendations for nonpharmacological pain management interventions to patients.

Change within a health care setting can be complicated. Quality improvement (QI) methods represent a way to improve patient care and outcomes, standardize processes, and create structure by using technology, education, and training. Standardization is key when embarking on QI and can be accomplished by making behaviors systematic and align with evidence. Systematic behavior decreases the chances of randomness by having the same actions result in the same outcomes. Identifying the random or non-standard behavior is reviewed in a series of steps of the Plan-Do-Study-Act (PDSA) cycle. Each time the PDSA cycle is used, the behavior becomes standardized [8].

The PDSA method helps test a change that is implemented by breaking down each step of the process, evaluating the outcome, improving the process, and testing it again. The first step of PDSA is the Plan phase. Planning involves making an ‘aim statement’ by asking what the goal of the proposed change is, how one will know that that change made improvements, and evaluating any other changes needed to result in an improvement. In the Do phase, the plan is implemented. This phase requires data to be collected so that the proposed change can be studied in step three. The data can be captured and reviewed in run charts, flowcharts, or check sheets. In the Study phase, the change is explored through the data to determine if the plan made any improvements or if there are any trends to notice. Unintended consequences may appear and be studied to ensure that the change can be sustainable. Last, the Act phase allows reflection on the plan to see if it was successful and to make it standard by using the new process regularly. If the data collected show a change is needed, then the PDSA cycle is started again. The PDSA cycle is ongoing as improvements are needed over time.

Quality improvement projects are ideal for implementing change within a hospital setting as they can empower clinicians and those at the front line of work. The PDSA cycle is driven by those invested in the outcome, and those individuals can become agents of change throughout the process [9]. In addition, the systematic nature of quality improvement can allow for small, purposeful changes in the implementation process. The aim of the current QI project was to increase the use of nonpharmacologic pain management strategies for patients in one unit of the inpatient setting within a pediatric East Coast hospital with pain scores of 5 or more by 20% over a 6-month period. This project was a pilot project with the intention of gathering data to better understand and improve the awareness and referral of nonpharmacological pain management services. It was hypothesized that the referrals for nonpharmacological pain management techniques would increase as a result of implementing the QI project. Although a long-term goal of the project is to reduce pain for hospitalized youth, the inconsistent use of nonpharmacological pain management strategies leads to significant difficulty in making conclusions about improvements in pain and the role of these techniques in pain management. Thus, a first step for the institution was to increase the use and consistency of nonpharmacological pain management strategies to then be in a better position to make inferences regarding changes in pain.

## 2. Materials and Methods

The project was approved as a Quality Improvement Initiative through the authors’ institutional IRB review committee in November 2021. The planning phase of the project took place from August 2021–November 2021, the PDSA cycles were implemented from November 2021–February 2022. Pre-QI baseline data was gathered in August and September 2021.

**Figure 1:**
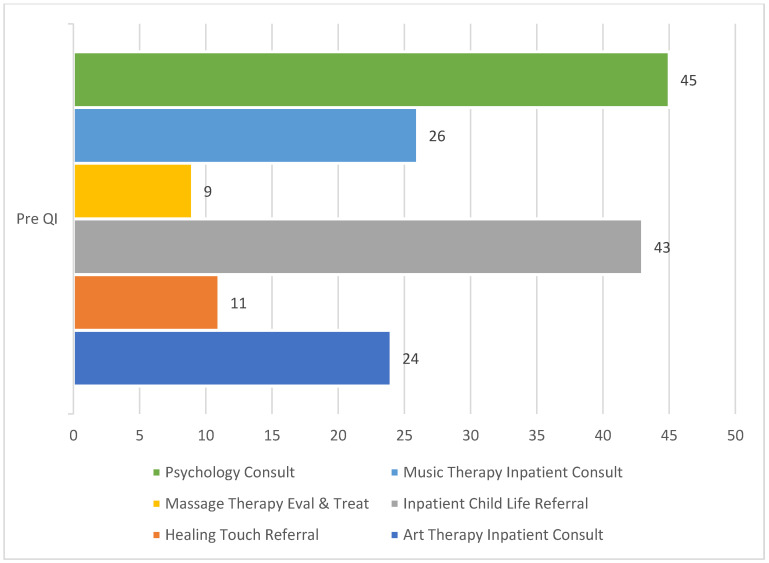
Referrals for nonpharmacological pain management strategies for the eight weeks prior to beginning the change cycles of the QI project.

**Figure 2:**
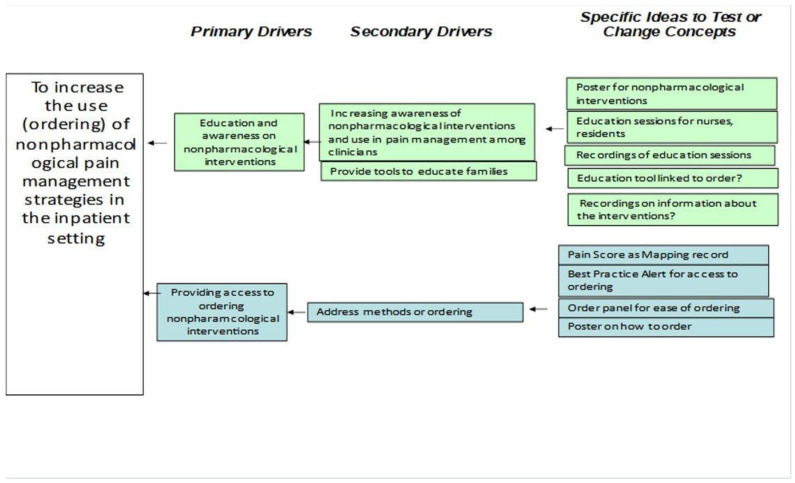
Driver diagram.

At the time of this quality improvement project, there were seven different nonpharmacological strategies available within the hospital system. These included Music Therapy, Art Therapy, Massage Therapy, Yoga, Healing Touch, Child Life, and Psychology. Despite the availability of these strategies, actual use within the inpatient setting was low across patients (see Figure 1). It was often observed that multiple strategies were offered to the same patient and some patients were not offered any nonpharmacological pain management strategies. A quality improvement project team was formed including representatives from the hospital’s chronic pain team, integrative medicine department, nursing, pharmacy, and psychology departments. A cause-and-effect diagram was completed in order to explore potential causes for the underuse of these strategies. This resulted in two primary drivers (see Figure 2): lack of education and awareness of the services within the hospital system and clinicians not having access to ordering the interventions for patients. Utilizing a driver diagram, several change ideas were identified for implementation in the change cycles. Only one of the inpatient units was selected, rather than generalizing the project to the entire hospital system.

### 2.1. Interventions

**Figure 3:**
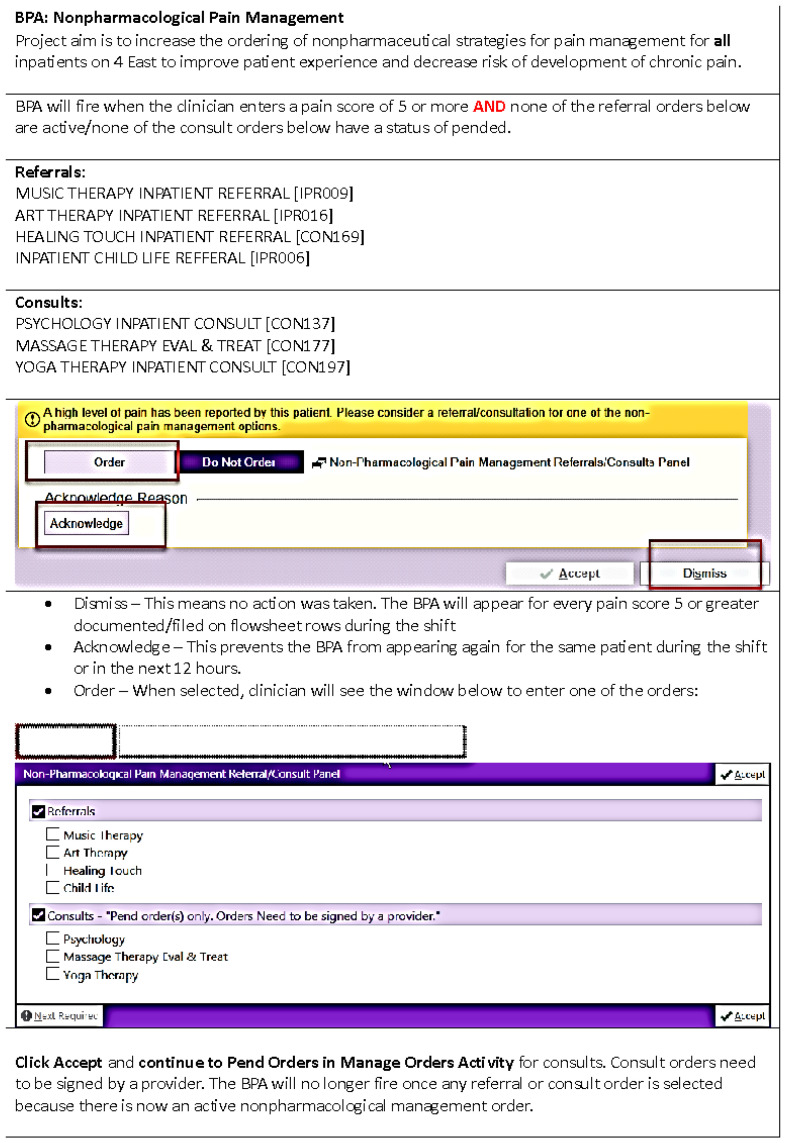
Best practice alert job aid.

Based on brainstorming within the driver diagram, four different interventions were identified, and the team developed the PDSA cycles in order to test each change idea. Each new intervention was implemented every three weeks. For the first intervention, a flyer was created for nursing and physician staff that included each nonpharmacological strategy, a few words on why it may be helpful for youth reporting pain during the admission, and information on how to order each strategy. These were distributed on the inpatient floor and posted in touchdown spaces as well as break rooms. For the second cycle, one of the team members gave a presentation to the nursing staff that included detailed information about the purpose of the project, details on each nonpharmacological pain management strategy, and indications for use including when to offer to youth reporting pain. This presentation was recorded. For the third cycle, two members of the team conducted a live version of the above-mentioned presentation for medical residents and fellows working on the inpatient unit. In addition, the prerecorded educational video of about 10 min in length was distributed to all nursing staff on the inpatient unit via email. In addition, two nurse members of the project team met individually with nurses on the unit and attended nursing daily huddles on this particular unit for three days to answer questions about the project and highlight the importance of utilizing nonpharmacological pain management strategies within the unit. The final intervention included the development of a best practice alert (BPA) within the electronic health record management system (Epic). Overall, a BPA is an automated alert that is embedded in the hospital’s Electronic Health Record (EHR). These alerts can disrupt routine behaviors by providing a reminder or clinical guideline at either a predetermined time point or when certain criteria are triggered. For the current project, a BPA was developed to trigger a message after a patient indicated a level 5 or higher pain score after two separate vital checks. Please see Figure 3 for the BPA Job Aid.

Given that the QI project was implemented within the workflow on the inpatient floor, and no information was gathered on the patients referred to the various nonpharmacological pain management strategies, no participant recruitment was completed. Medical professionals attended didactics as planned.

#### 2.1.1. Outcome Measure

To measure the outcome of each intervention cycle, the total number of orders for each of the nonpharmacological strategies was obtained from the EHR. This number was totaled for each week of the project.

#### 2.1.2. Balance Measure

A brief survey for counterbalance measures was sent to nurses on the inpatient unit and to the therapists that were providing the nonpharmacological services within the project. The goal was to ensure that the project did not add an extra burden to those involved in carrying out the change cycles. The survey included five questions about the nurse’s/therapist’s general level of stress, estimated number of consults placed/received, number of patients who refused the consult, and if the overall project was a benefit or an additional burden in day-to-day activities. Surveys were sent electronically, weekly via email with a REDCap [10,11] link to complete the survey.

For data analysis, the total number of orders for the nonpharmacological pain management strategies were tracked weekly throughout the project timeline. An annotated run chart was created in Microsoft Excel 2021 (Microsoft 365) to reflect the number of orders and change cycles. Data from the nursing and therapist questionnaires were analyzed using Microsoft Excel 2021 (Microsoft 365). SQUIRE 2.0 guidelines were utilized as a template for the project [12].

## 3. Results

**Figure 4:**
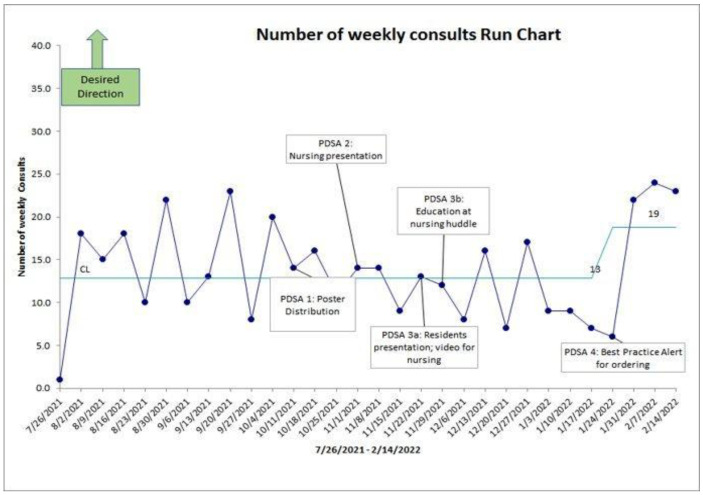
Annotated run chart.

For the eight weeks prior to implementing the first PDSA cycle, there was an average of 15.5 referrals for all nonpharmacological pain management strategies. As observed within the annotated run chart (Figure 4), orders for nonpharmacological pain management strategies were low and inconsistent prior to the quality improvement project and change cycles. During the first three PDSA cycles, a similar pattern was noted. Following the first PDSA cycle, the average number of referrals received was 13.75 per week. Between cycles two and three, the average number of referrals per week was 12. Finally, between cycles three and four, the average orders received for nonpharmacological pain management strategies was 9.88 per week. During the process, it was observed that the first three cycles were primarily education focused. For the final change cycle, the focus of the BPA was to provide access to ordering the strategies. An average of 23 orders per week were received for nonpharmacological pain management strategies for the three weeks following the implementation of the BPA. This represents a 42% increase from baseline (using eight weeks) and a 50% increase in orders for nonpharmacological pain management strategies on average (using data following PDSA Cycle 1). Of note, there were no orders received for yoga during the entire length of the project.

**Figure 5:**
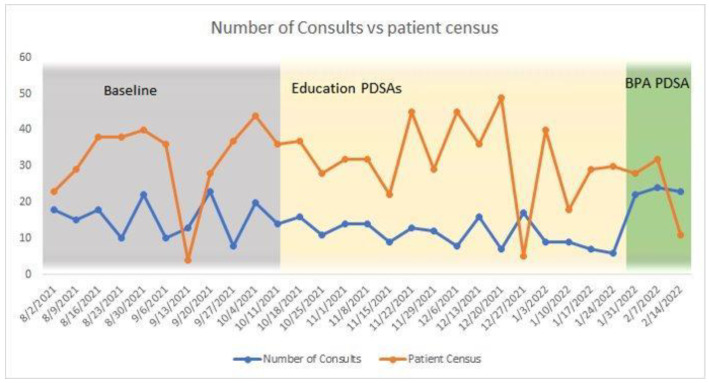
Patient census and number of nonpharmacological pain management strategy orders.

Patient census was tracked as a consideration that could affect the number of orders placed. Over the course of the baseline data (prior to PDSA cycles) and throughout the quality improvement project an average of 31.5 patients were admitted per week on the specific inpatient unit utilized for the project. As seen in Figure 5, patient census was generally high and was not correlated with the number of orders placed (r = −0.207, *p* = 0.396).

**Figure 6:**
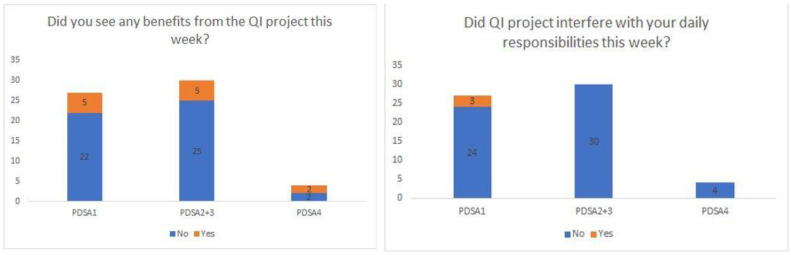
Nurses’ and therapists’ reports of benefit and interference.

Unfortunately, only 61 responses were received via the survey sent to nurses and therapists throughout the entire length of the project. Only two of the seven therapists responded to the survey during the project period. Overall, nurses and therapists reported a wide range of levels of stress throughout the project ranging from 5 to 100 (0 no stress, 100 most stressed) with an average of 53. Most nurses and therapists did not report a benefit from the project, although the majority of the nurses and therapists did not believe that the project interfered with their daily responsibilities during any point of the PDSA cycles (see Figure 6).

## 4. Discussion

Overall, the project revealed that education alone did not result in increases in the use of nonpharmacological pain management strategies within an inpatient hospital setting. In fact, only a BPA triggered through the EHR led to an increase. This is valuable knowledge when thinking about future projects aiming to increase utilization of nonpharmacological strategies. These results were not initially expected as it was hypothesized that providing education about the use of nonpharmacological pain management strategies would be enough to lead to an increase in orders. Castellano-Tejedor [13] highlight that nonpharmacological interventions such as cognitive behavioral therapy and yoga do contribute to improved pain outcomes. Patients can benefit from nonpharmacological pain management strategies through empowerment, goal setting, and relaxation; thus, future research on how to better embed nonpharmacological pain management strategies into inpatient settings is necessary and ideal for improved outcomes.

Several lessons were learned during this quality improvement project. First, including staff from the inpatient unit on the QI team is incredibly helpful in encouraging change and determining any barriers to change. At the time of the project, the healthcare system was struggling with retaining nursing staff and several nurse champions left the organization in the middle of the project. Nurse retention is a global concern and the American Nurses Association [14] has outlined several strategies to retain and engage nurses. These include career advancement pathways and autonomy. Second, relying on education only, whether through posters, in-person or virtual education, or participation in huddles was not successful in improving the rate of nonpharmacological pain management strategies for youth experiencing pain while hospitalized. The authors suspect that this lack of success may have been because these education interventions were not reaching all nurses on the unit. Last, utilizing functions within the EHR can be the most effective way to increase orders for other pain management interventions. This intervention allows for nurses to be prompted to place the orders for the nonpharmacologic strategies that they are authorized to request or inquire about these interventions from the medical team which led to these orders being placed.

Given that the BPA was so successful in improving orders for most of the nonpharmacologic pain management strategies, future efforts to include nonpharmacological pain management strategies within inpatient settings may consider adding these order sets to existing care delivery models in other institutions. For example, including these orders within order sets traditionally designed for pain medication may serve as a reminder to clinicians to consider the biopsychosocial model in managing all types of pain. Ng and colleagues [15] reflected on ways to reduce the burden of best practice alerts by categorizing various similar order sets, which lead to an increase in follow-through with best practice alerts from nurses in a hospital system in Singapore. Another consideration is to include nonpharmacological pain management strategies in the nursing and physician admission assessments within the emergency department or upon arrival to the admitting floor. Including documentation in the admission database with links for orders for the nonpharmacologic pain management strategies may contribute to an increase in thoughtfulness and orders. It will also allow for these interventions to be implemented sooner in the course of hospitalization, which will likely improve their success rate and their acceptance from families and patients.

One consideration is that the combination of the education and access to ordering the nonpharmacological pain management strategies is what lead to the overall change in orders by the end of the quality improvement project. Nilson and colleagues [16] note that changes in healthcare are more apt to succeed if personnel are provided a chance to influence, prepare, and recognize the value of the change. In a fast-paced hospital setting, it is often difficult to allow time for reflection on changes. Direct evidence and observations on the benefit to the patients and staff is also helpful. Utilizing the PDSA cycle is optimal in promoting change within a healthcare setting due to the intentional pauses, data gathering, and review of each cycle. McCann and Robson [17] acknowledge the complexity of the healthcare system and the need to act through a quality improvement lens. In their chapter, the authors indicate that a systems approach is necessary to engage in change.

Additionally, there needs to be careful consideration on how to provide education on the importance and use of nonpharmacological pain management strategies. Continuing education programs for nurses and other healthcare professionals should be interactive, engaging, and allow for time to process the new information and apply it [18]. For example, a hospital system could develop and introduce standardized education within nursing orientation, new clinician hospital orientation, and orientation for new medical residents and fellows about the nonpharmacological pain management strategies that are available at that specific hospital and how to order these for patients in the inpatient setting. A nurse or physician champion could then follow up on these educational offerings to check in on questions about the use of these strategies, the order process, and any barriers that may have been observed within practice. Continuing to track orders and adjust education would be an integral part of the process. In the current study, yoga was not ordered as one of the nonpharmacological pain management strategies. Although this was not directly addressed within the QI project, exploration as to why yoga was not ordered could have helped refine the education provided and potentially lead to an increase in orders by the end of the project.

The quality improvement process was very beneficial and provided a wide range of data to aid with subsequent PDSA cycles. The team was able to adapt between each PDSA cycle. For example, the original plan did not include attending huddles or providing a recorded version of the educational materials. Input from the nurses and other staff allowed for this adaptation. While it may not have influenced the overall outcome, involvement from staff allowed implementation of the BPA to be more successful, and these staff were intricately involved in the planning process. Knowledge of the nursing flow allowed for quick dissemination of the BPA job aid, likely leading to the success that this intervention had in increasing orders for nonpharmacological pain management strategies.

Balancing measures were utilized via surveys to ensure that staff were not overburdened, especially those providing the nonpharmacological pain management strategies. Unfortunately, few of these surveys were completed throughout the project timeline. This made it difficult to understand if there was an increase in burden to the nurses or the therapists with the implementation of this project. Of those who completed the survey, very few reported burden or benefit. In the future, it will be important to determine the best way to assess burden and benefit from those implementing the interventions and conducting the change behaviors.

One limitation of this QI project was the focus on increasing the use of nonpharmacological pain management strategies. Because of the structure of the data obtained, the reason for the orders was not obtained, making it difficult to know if the order was related to pain management or some other indication. Along those same lines, it is difficult to determine from the data if the services were offered to multiple patients or if only a few select patients received multiple referrals for the strategies. Additionally, the impact on pain, pain reporting, and medication use was not measured as part of the project. Given that the project was guided by the assumption that nonpharmacological strategies would be helpful, no data about outcomes of pain were assessed. The ultimate goal is to ensure consistent use of nonpharmacological pain management strategies in order to then explore the role on pain and medication use. If inconsistencies remain, then it will be difficult to make inferences on if and how these strategies may help with reducing pain. In addition, this project was implemented on a very small scale only (one unit at one hospital); therefore, future research should replicate these findings in a larger project for all units within the hospital and consider adding pain and medication use as additional outcome variables. Lastly, this project occurred during the winter of 2021 into 2022 when many of the COVID-19 restrictions were still in place within the hospital and there was a large surge of acute COVID-19 cases, which may have limited the ability of some therapies because of restrictions placed by hospital policy and infection control procedures. There was also a large nursing turnover rate within the hospital at this time, potentially affecting the results.

## 5. Conclusions

It is clear that continued collaboration with all partners involved in implementing this type of quality improvement project is necessary for success. In the future, when constructing pain management order sets within the EHR, both pharmacological and nonpharmacological pain management strategies should be included. A best practice alert, or BPA, would likely be the best means to increase the use of nonpharmacological pain management techniques in the inpatient setting for youth experiencing pain. At the very least, it will promote more consistent referral of patients to these nonpharmacological services. Using this biopsychosocial approach to all pain management, no matter the cause or clinical scenario, will not only improve acute pain management but potentially also reduce the risk of chronic pain. The patient and family will be taught important generalizable techniques that will help manage any pain they may encounter in the future.

## Data Availability

The data presented in this study are available on request from the corresponding author. The data are not publicly available because of privacy restrictions.
